# A review of myrmecophilous mites of the family Microdispidae (Acari, Heterostigmatina) of Western Siberia

**DOI:** 10.3897/zookeys.454.8709

**Published:** 2014-11-12

**Authors:** Alexander A. Khaustov

**Affiliations:** 1 Tyumen State University, Tyumen, Russia

**Keywords:** Acari, Heterostigmatina, systematics, phoresy, ants, Western Siberia

## Abstract

Five species of myrmecophilous microdispid mites (Acari: Microdispidae) are recorded from Western Siberia, Russia. *Unguidispus lasii* Kurosa, 1979, *Unguidispus japonicus* Kurosa, 1979, *Caesarodispus minutus* (Sevastianov, 1981), and *Caesarodispus samsinaki* (Mahunka, 1967), **comb. n.** are reported from Russia for the first time. *Unguidispus polyctenus* (Sevastianov, 1969) and *Caesarodispus samsinaki* are redescribed. The keys to species of the genera *Unguidispus* Mahunka, 1970 and *Caesarodispus* Mahunka, 1977 are provided.

## Introduction

The family Microdispidae Cross, 1965 (Acari: Pygmephoroidea) includes 18 genera and about 115 described species (Hajiqanbar and Hosseininaveh 2014). All representatives from the genera *Unguidispus* Mahunka, 1970, *Caesarodispus* Mahunka, 1977, *Myrmecodispus* Cross, 1965, *Reductodispus* Mahunka, 1977, *Perperipes* Cross, 1965, and *Glyphidomastax* Cross, 1965 are associated with various ants (Hymenoptera: Formicidae) (Hajiqanbar and Hosseininaveh 2014). Most of microdispid mites are fungivorous, but the species from the genera *Perperipes* and *Glyphidomastax*, associated with army ants, probably feed on their larvae and/or eggs (Kaliszewski et al. 1995). During the study of myrmecophilous mites of Western Siberia we found five species of the family Microdispidae belonging to the genera *Unguidispus* Mahunka, 1970 and *Caesarodispus* Mahunka, 1977. The main goal of this paper is to redescribe the poorly known species *Unguidispus polyctenus* (Sevastianov, 1969) and *Caesarodispus samsinaki* (Mahunka, 1967), comb. n. and provide new records of myrmecophilous microdispid mites from Western Siberia, as well as the keys to world species of the genera *Unguidispus* and *Caesarodispus*.

## Materials and methods

Mites were collected from ants or ant nests and mounted in Hoyer’s medium. The terminology of idiosoma and legs follows Lindquist (1986); the nomenclature of subcapitular setae and the designation of cheliceral setae follow Grandjean (1944, 1947), respectively. The system of Pygmephoroidea follows Khaustov (2004, 2008). All measurements are given in micrometers (μm). For leg chaetotaxy the number of solenidia is given in parentheses. The studied material is deposited in the mite collection of the Tyumen State University Museum of Zoology, Tyumen, Russia. SEM photographs were made with the aid of JEOL–JSM-6510LV SEM microscope.

## Systematics

### Family Microdispidae Cross, 1965

#### Genus Unguidispus

Taxon classificationAnimaliaProstigmataMicrodispidae

Mahunka, 1970

##### Type species.

*Unguidispus stammeri* Mahunka, 1970, by original designation.

##### Diagnosis.

**Female.** Gnathosoma dorsally with 2 pairs of setae. Pharyngeal pumps 1 and 3 vestigial, pharyngeal pump 2 large, transversely striated. Prodorsum usually almost completely covered by tergite C. Cupules *ia* and *ih* small, round. Two pairs of pseudanal setae present (*ps*_2_ absent). Posterior margin of posterior sternal fig entire. Leg I distinctly shorter than leg II. Tibiotarsus with well-developed claw; eupathidia *tc*’-*tc*” situated on clear pinnaculum; setae *s* of tibiotarsus I present. Trochanter IV anterodorsally with short spine-like process.

The genus *Unguidispus* currently includes six species distributed in the Palaearctic region: *Unguidispus stammeri*, *Unguidispus polyctenus* (Sevastianov, 1969), *Unguidispus contematosus* Sevastianov, 1981, *Unguidispus okumurai* Kurosa, 1979, *Unguidispus japonicus* Kurosa, 1979, and *Unguidispus lasii* Kurosa, 1979. All species of the genus *Unguidispus* phoretic on ants of the genera *Formica* L. and *Lasius* Fabricius (Hymenoptera: Formicinae), or inhabit their nests (Hajiqanbar and Hosseininaveh 2014; Kurosa 1979).

#### Unguidispus polyctenus

Taxon classificationAnimaliaProstigmataMicrodispidae

(Sevastianov, 1969)

Figs 1 , 2 , 3 , 4

Piniphorus polyctenus Sevastianov, 1969, p. 68, fig. 2.
Xystrorostrum polyctenus : Mahunka 1970a: 165.
Unguidispus polyctenus : Mahunka 1970b: 282.

##### Redescription.

**Female** Length of idiosoma 190–205, width 120–135.

**Figure 1.**
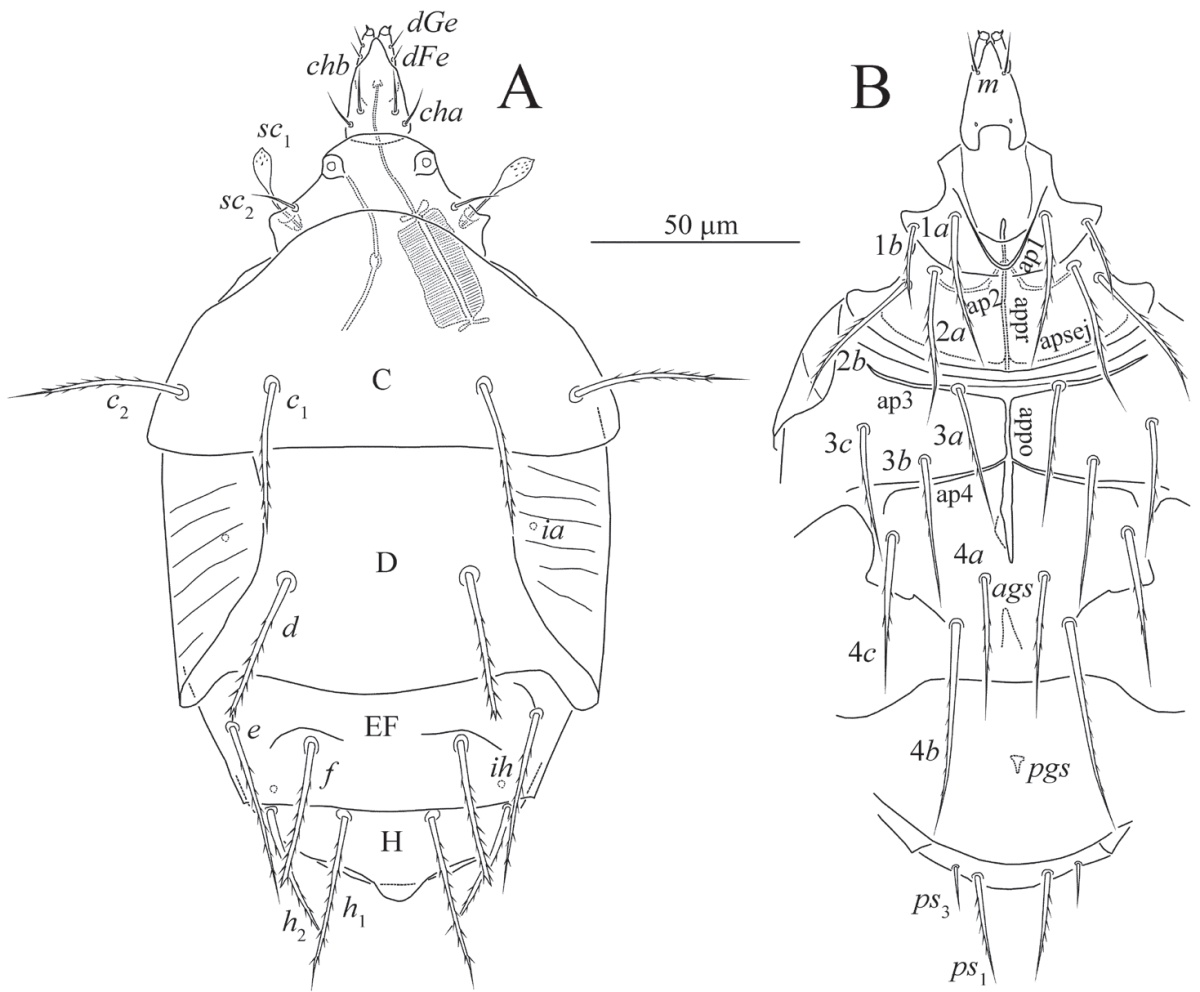
*Unguidispus polyctenus* (Sevastianov, 1969), female: **A** dorsum of the body **B** venter of the body.

*Gnathosoma* (Figs 1, 4E, 4F). Gnathosomal capsule beak-like, about 1.5 times longer than its width. Dorsally with two pairs of smooth subequal setae (*cha*, *chb*). Dorsal median apodeme absent. Ventral gnathosoma with one pair of subcapitular setae *m* and a pair of oval pits situated posteromedial to bases of *m*. Palps with setae *dFe* and *dGe* dorsolaterally. Setae *dGe* slightly longer than *dFe*. Ventral palpal structures not evident even on SEM photos (Fig. 4F). Palps terminated with a relatively long and thin tibial claw (Fig. 4F).

*Idiosomal dorsum* (Figs 1A, 4A). Prodorsum almost completely covered by anterior margin of tergite C, with one pair of setae *sc*_2_, one pair of clavate and weakly barbed trichobothria *sc*_1_ with pointed apex, and one pair of large round stigmata. All dorsal shields smooth. All dorsal setae distinctly barbed; setae *d* and *f* blunt-ended, other dorsal setae pointed. Posterior margin of tergite C straight; posterior margin of tergite H with tongue-like elongation medially. Cupules *ia* on tergite D and *ih* on tergite H small, round. Lateral shields covering lateral parts of tergite D with distinct sclerotized lines (Fig. 4B). With a pair of arch-like structures anteriorly to setae *f* and a pair of oblique ridges connected to bases of setae *e* (Fig. 4C). Length of dorsal setae: *sc*_2_ 12–14, *c*_1_ 35–38, *c*_2_ 40–42, *d* 36–38, *e* 44–45, *f* 35–37, *h*_1_ 40–45, *h*_2_ 35–37. Distances between setae: *sc*_2_–*sc*_2_ 37–40, *c*_1_–*c*_1_ 47–50, *c*_1_–*c*_2_ 22–24, *d*–*d* 41–43, *e*–*f* 18–20, *f*–*f* 35–38, *h*_1_–*h*_1_ 19–21, *h*_1_–*h*_2_ 18–21.

*Idiosomal venter* (Figs 1B, 4D). All ventral figs smooth. All ventral setae pointed and barbed, except smooth *ps*_3_. Apodemes 1 (ap1) weakly developed and joined with prosternal apodeme (appr); apodemes 2 (ap2) well developed, arch-like, fused with appr; prosternal and sejugal (apsej) apodemes well developed; apodemes 3 (ap3) well sclerotized. Apodemes 4 (ap4) well sclerotized and long, apodemes 5 absent. Posterior margin of posterior sternal fig slightly convex in middle part. Posterior margin of aggenital fig rounded. Anterior genital sclerite (ags) bell-like, posterior genital sclerite (pgs) very small, triangular. Length of ventral setae: 1*a* 35–37, 1*b* 21–23, 2*a* 33–36, 2*b* 36–40, 3*a* 34–36, 3*b* 35–37, 3*c* 36–38, 4*a* 36–38, 4*b* 52–55, 4*c* 39–41, *ps*_1_ 24–26, *ps*_3_ 9–11.

**Figure 2.**
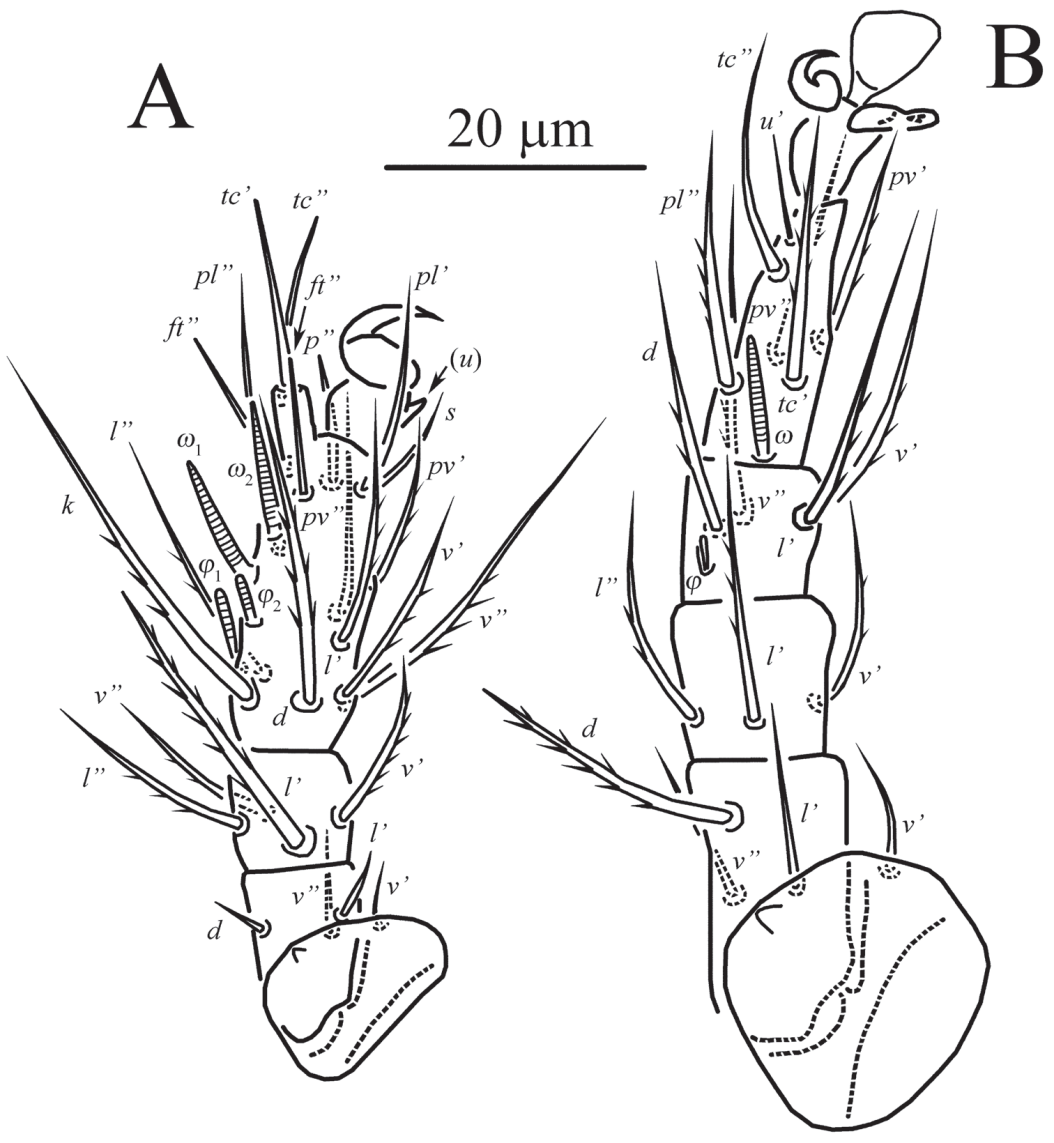
*Unguidispus polyctenus* (Sevastianov, 1969), female: **A** leg I **B** leg II.

**Figure 3.**
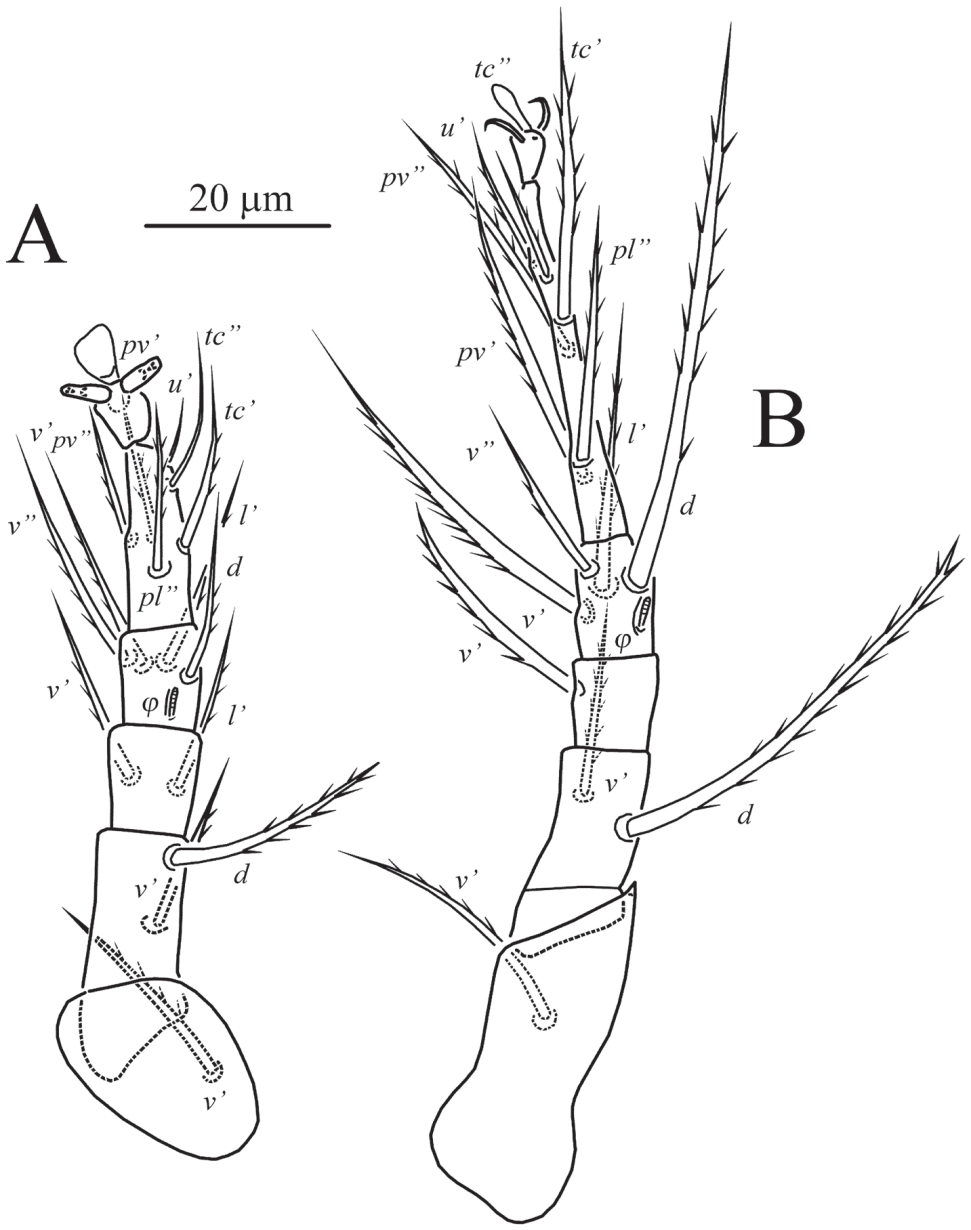
*Unguidispus polyctenus* (Sevastianov, 1969), female: **A** leg III **B** leg IV.

**Figure 4.**
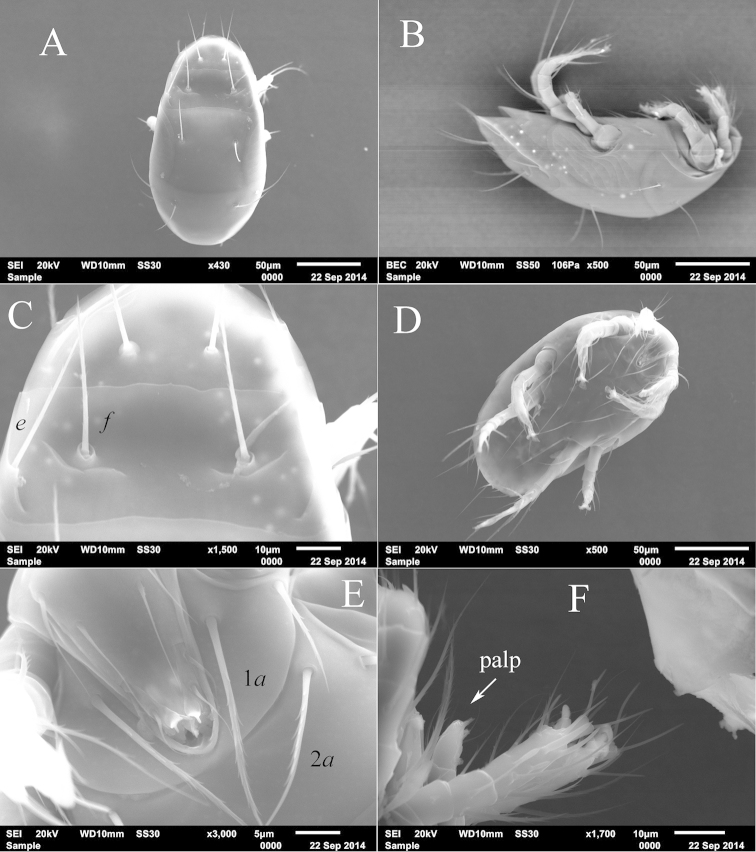
*Unguidispus polyctenus* (Sevastianov, 1969), female, SEM photos: **A** dorsum of the body **B** lateral view of the body **C** tergites EF and H **D** venter of the body **E** gnathosomal capsule **F** legs I and distal part of gnathosoma laterally.

*Legs* (Figs 2–3, 4F). Leg I (Figs 2A, 4F) distinctly shorter and thinner than leg II. Setal formula: 1–3–4–16(4). Tibiotarsus not thickened, with well-developed terminal claw situated on distinct pretarsus, tip of the claw thin. Length of solenidia *ω*_1_ 11–12 = *ω*_2_ 11–12 > *φ*_1_ 6–7 = *φ*_2_ 6–7; *ω*_1_ and *ω*_2_ finger-shaped, *φ*_2_ baculiform, *φ*_1_ clavate. Setae (*u*) fused into structure opposing to tarsal claw. Leg II (Fig. 2B). Setal formula: 1–3–3–4(1)–6(1). Tarsus with sickle-like, padded claws and large empodium. Solenidion *ω* 9–10, finger-shaped, solenidion *φ* 3–4 weakly clavate. Seta *dFe* distinctly blunt-ended. Leg III (Fig. 3A). Setal formula: 1–2–2–4(1)–6. Claws of same shape as on tarsus II. Solenidion *φ* 3–4 weakly clavate. Seta *dFe* distinctly blunt-ended. Leg IV (Fig. 3B). Setal formula: 1–2–1–4(1)–6. Tarsus long and thin, pretarsus short, with two small simple claws and small empodium. Solenidion *φ* 3–4, weakly clavate. Seta *dFe* distinctly blunt-ended.

**Male** and **larva** unknown.

##### Material examined.

Eight female paratypes, **Ukraine**, Khmelnitsk Province, vicinity of settlement Chemerovtsy, on ants *Formica polyctena* Forster, 23 July 1966, coll. V.D. Sevastianov; 3 females, **Russia:** Tyumen Province, vicinity of Tyumen, 57°04'03"N, 65°04'12"E, on ants *Formica rufa* L., 17 August 2014, coll. A.A. Khaustov; 15 females, **Russia**, Tyumen Province, vicinity of Tyumen, 57°13'43.6"N, 65°28'28.4"E, on ants *Formica polyctena*, 14 September 2014, coll. A.A. Khaustov.

##### Distribution and hosts.

This species was described from Western Ukraine from ants *Formica polyctena* Forster (Sevastianov 1969). It was also recorded in northwestern Russia (Sevastianov 1978), Austria (Mahunka 1970b) from ants *Formica rufa* L., and in Hungary from ant nest (Mahunka 1987).

##### Remarks.

Sevastianov (1969) placed this species in the genus *Piniphorus* Sevastianov, 1969. Mahunka (1970a) transferred it to *Xystrorostrum* Mahunka, 1968 (Neopygmephoridae) which he considered as a senior synonym of *Piniphorus*. The same year Mahunka (1970b) moved this species to the microdispid genus *Unguidispus* Mahunka, 1970. In spite of this, Sevastianov (1978) retained it in the neopygmephorid genus *Xystrorostrum*.

The original description of *Unguidispus polyctenus* of Sevastianov (1969) is incomplete, thus I made a redescription of this species. The present redescription of *Unguidispus polyctenus* is based mainly on material from Western Siberia. The female paratypes available for this study are found in bad condition, yet sufficient to prove their identity with mite specimens from Western Siberia.

#### Unguidispus lasii

Taxon classificationAnimaliaProstigmataMicrodispidae

Kurosa, 1979

Unguidispus lasii Kurosa, 1979, p. 66, figs 5–6.

##### Material examined.

Six females, **Russia:** Tyumen Province, vicinity of Tyumen, 57°04'03"N, 65°04'12"E, on ants *Lasius niger* L., 17 August 2014, coll. V.M. Salavatulin; 5 females, Russia: Tyumen Province, vicinity of Tyumen, 57°09'55"N, 65°27'32"E, on ants *Lasius niger*, 31 August 2014, coll. A.A. Khaustov.

This species was described from Japan where it was found in the nests of ants *Lasius niger* and *Lasius hayashi* Yamauchi and Hayashida (Kurosa 1979). I recorded phoresy of this species on *Lasius niger* in Western Siberia. This is a new record for the fauna of Russia.

#### Unguidispus japonicus

Taxon classificationAnimaliaProstigmataMicrodispidae

Kurosa, 1979

Unguidispus japonicus Kurosa, 1979, p. 64, figs 1–2.

##### Material examined.

Five females, **Russia:** Tyumen Province, vicinity of Tyumen, 57°04'03"N, 65°04'12"E, on ants *Lasius niger*, 17 August 2014, coll. V.M. Salavatulin. 2 females, **Russia:** Tyumen Province, vicinity of Tyumen, 57°09'55"N, 65°27'32"E, on ants *Lasius niger*, 31 August 2014, coll. A.A. Khaustov.

This species was described from Japan from the nests of ants *Lasius niger* (Kurosa 1979). I recorded phoresy of this species on *Lasius niger* in Western Siberia. This is a new record for the fauna of Russia.

#### Key to world species of the genus *Unguidispus* (females)

**Table:** 

1	All dorsal hysterosomal setae unmodified	**2**
–	At least setae on tergites C and D flattened, widened distally, and heavily barbed	**3**
2	Setae *c*_1_, *d*, and *f* thin and smooth, without arch-like ridges anteriorly to setae *f*	***Unguidispus okumurai* Kurosa, 1979.** Japan. On *Lasius hayashi*
–	All dorsal hysterosomal setae strongly barbed, with arch-like ridges anteriorly to setae *f*	***Unguidispus polyctenus* (Sevastianov, 1969).** Ukraine, Austria, Hungary, Russia. On *Formica rufa*, *Formica polyctena*
3	Setae *h*_1_ flattened, widened distally and barbed	**4**
–	Setae *h*_1_ thin, smooth, pointed	***Unguidispus contematosus* Sevastianov, 1981.** Ukraine. On *Lasius fuliginosus*
4	Setae *e* distinctly thickened, subequal to or longer than *f*	**5**
–	Setae *e* not thickened, distinctly shorter than *f*	***Unguidispus lasii* Kurosa, 1979.** Japan, Russia. On *Lasius niger*, *Lasius hayashi*
5	Setae *h*_2_ pointed, setae *d* distinctly thicker than *f*, trichobothria spherical	***Unguidispus japonicas* Kurosa, 1979.** Japan, Russia. On *Lasius niger*
–	Setae *h*_2_ widened distally, setae *d* as thick as *f*, trichobothria pointed distally	***Unguidispus stammeri* Mahunka, 1970.** Hungary. In ant nest.

#### Genus Caesarodispus

Taxon classificationAnimaliaProstigmataMicrodispidae

Mahunka, 1977

##### Type species.

*Caesarodispus gaius* Mahunka, 1977, by original designation.

##### Diagnosis.

**Female.** Gnathosoma dorsally with two pairs of setae. Pharyngeal pumps 1 and 3 vestigial, pharyngeal pump 2 large, transversely striated. Prodorsum usually almost completely covered by tergite C. Cupules *ia* and *ih* small, usually round. Two pairs of pseudanal setae (*ps*_2_ absent). Posterior margin of posterior sternal fig entire. Leg I distinctly shorter than leg II. Tibiotarsus without claw; pinnaculum absent; setae *s* of tibiotarsus I present. Trochanter IV anterodorsally without spine-like process.

The genus *Caesarodispus* currently includes nine species distributed in the Holarctic region: *Caesarodispus gaius*, *Caesarodispus samsinaki* (Mahunka, 1967), comb. n., *Caesarodispus minutus* (Sevastianov, 1981), *Caesarodispus acuminatus* (Sevastianov, 1981), *Caesarodispus klepzigi* Khaustov & Moser, 2008, *Caesarodispus pusillus* Khaustov, 2009, *Caesarodispus brevipes* Mahunka, 1986, *Caesarodispus modestus* (Berlese, 1903), and *Caesarodispus shandizensis* Loghmani & Hajiqanbar, 2014. All species of the genus *Caesarodispus* phoretic on various ants, or inhabit their nests. Loghmani et al. (2014) discussed distribution and host specificity of the genus *Caesarodispus* and provided key to eight species.

#### Caesarodispus samsinaki

Taxon classificationAnimaliaProstigmataMicrodispidae

(Mahunka, 1967) comb. n.

Figs 5 , 6 , 7

Pygmephorus samsinaki Mahunka, 1967, p. 241, fig. 1.
Brennandania samsinaki : Mahunka 1972: 82.
Petalomium samsinaki : Sevastianov 1978: 37.

##### Redescription.

**Figure 5.**
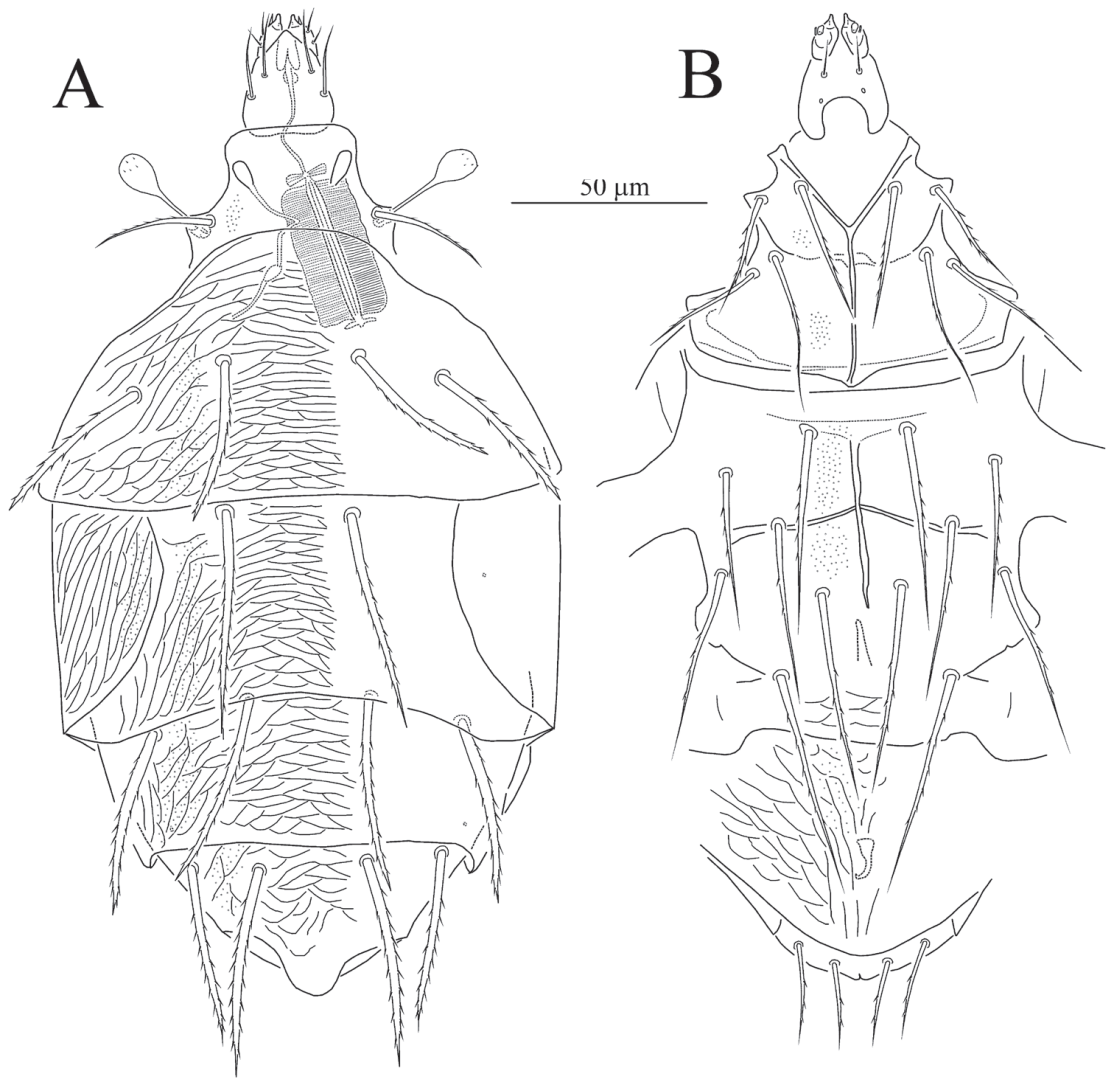
*Caesarodispus samsinaki* (Mahunka, 1967), comb. n., female: **A** dorsum of the body **B** venter of the body.

**Figure 6.**
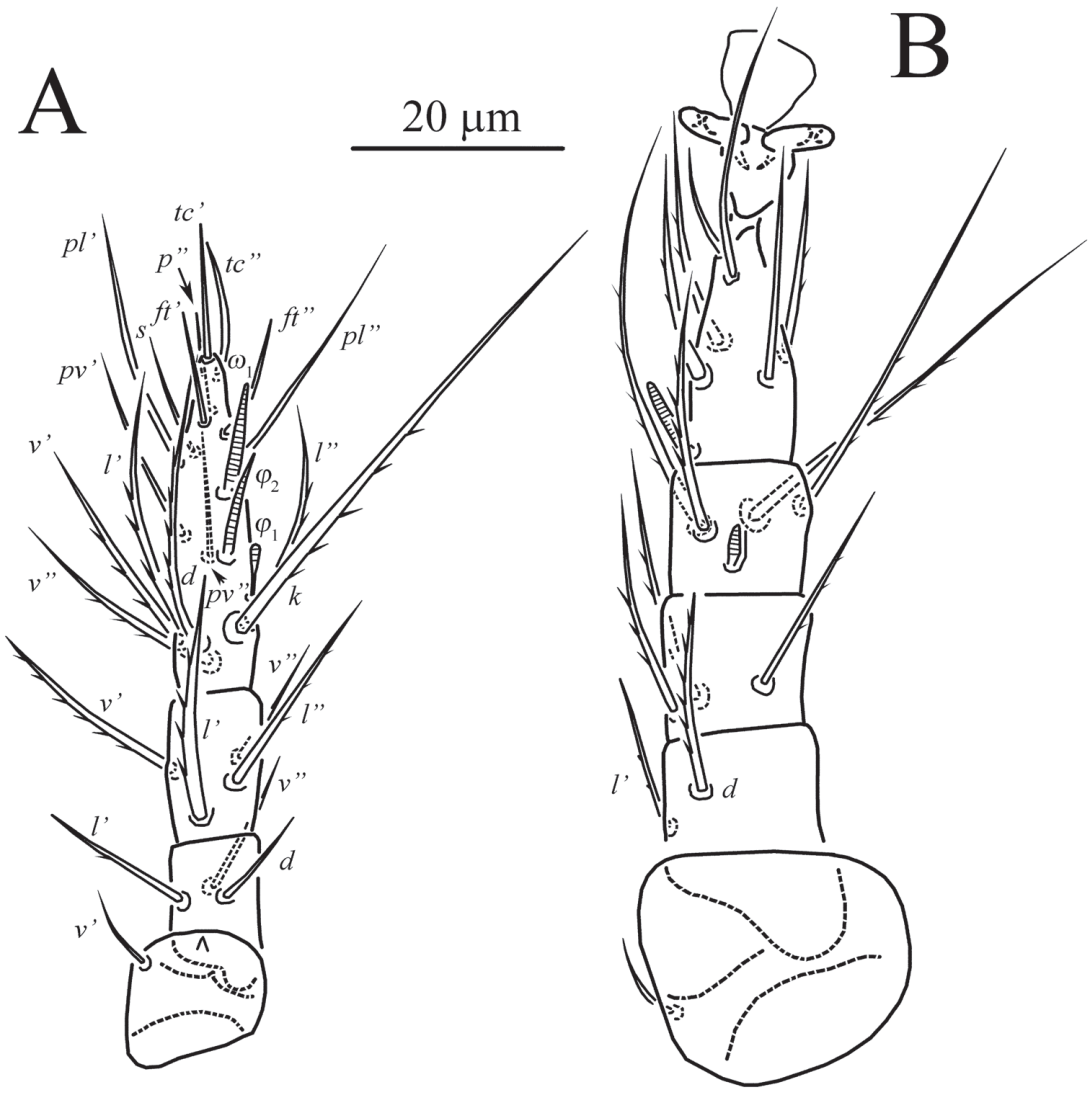
*Caesarodispus samsinaki* (Mahunka, 1967), comb. n., female: **A** leg I **B** leg II.

**Female.** Length of idiosoma 220, width 135. *Gnathosoma* (Figs 5–6). Gnathosomal capsule about as long as its width. Dorsally with two pairs of smooth setae (*cha*, *chb*). Setae *cha* slightly longer than *chb*. Dorsal median apodeme absent. Ventral gnathosoma with one pair of subcapitular setae *m* and a pair of oval pits situated posteromedial to bases of *m*. Palps short, with setae *dFe* and *dGe* dorsolaterally. Setae *dGe* slightly longer than *dFe*. Ventrally with tiny solenidion and accessory setigenous structure. Palps terminated with a relatively short and thick tibial claw. Palpal tibiotarsus laterally with small triangular translucent process. Pharyngeal pumps 1 and 3 small, vestigial; pharyngeal pumps II large, transversely striated (Fig. 5A).

*Idiosomal dorsum* (Fig. 5A). Tergite C covering only posterior part of prodorsum, which bears one pair of setae *sc*_2_, one pair of spherical and weakly barbed trichobothria *sc*_1_ and one pair of oval stigmata. Prodorsal shield with sparsely distributed small dimples. All hysterosomal tergites distinctly reticulated and with sparsely distributed small dimples. All dorsal setae lanceolate, sparsely barbed. Posterior margin of tergite C straight; posterior margin of tergite H with tongue-like elongation medially. Cupules *ia* on tergite D and *ih* on tergite H very small, round. Length of dorsal setae: *sc*_2_ 32, *c*_1_ 42, *c*_2_ 44, *d* 58, *e* 47, *f* 50, *h*_1_ 54, *h*_2_ 46. Distances between setae: *sc*_2_–*sc*_2_ 43, *c*_1_–*c*_1_ 35, *c*_1_–*c*_2_ 24, *d*–*d* 31, *e*–*f* 25, *f*–*f* 30, *h*_1_–*h*_1_ 29, *h*_1_–*h*_2_ 19.

Idiosomal venter (Fig. 5B). All ventral figs with small dimples. Posterior part of posterior sternal fig and aggenital fig reticulated. All ventral setae pointed and barbed. Ap1 well-developed and joined with appr; ap2 thin, arch-like, fused with appr; appr and apsej well developed; ap3 indistinct. Ap4 well sclerotized and long, apodemes 5 absent. Posterior margin of posterior sternal fig slightly convex in middle part. Posterior margin of aggenital fig rounded. Ags bell-like, pgs elongate, subtriangular. Length of ventral setae: 1*a* 35, 1*b* 32, 2*a* 42, 2*b* 39, 3*a* 52, 3*b* 47, 3*c* 40, 4*a* 54, 4*b* 60, 4*c* 47, *ps*_1_ 23, *ps*_3_ 24.

**Figure 7.**
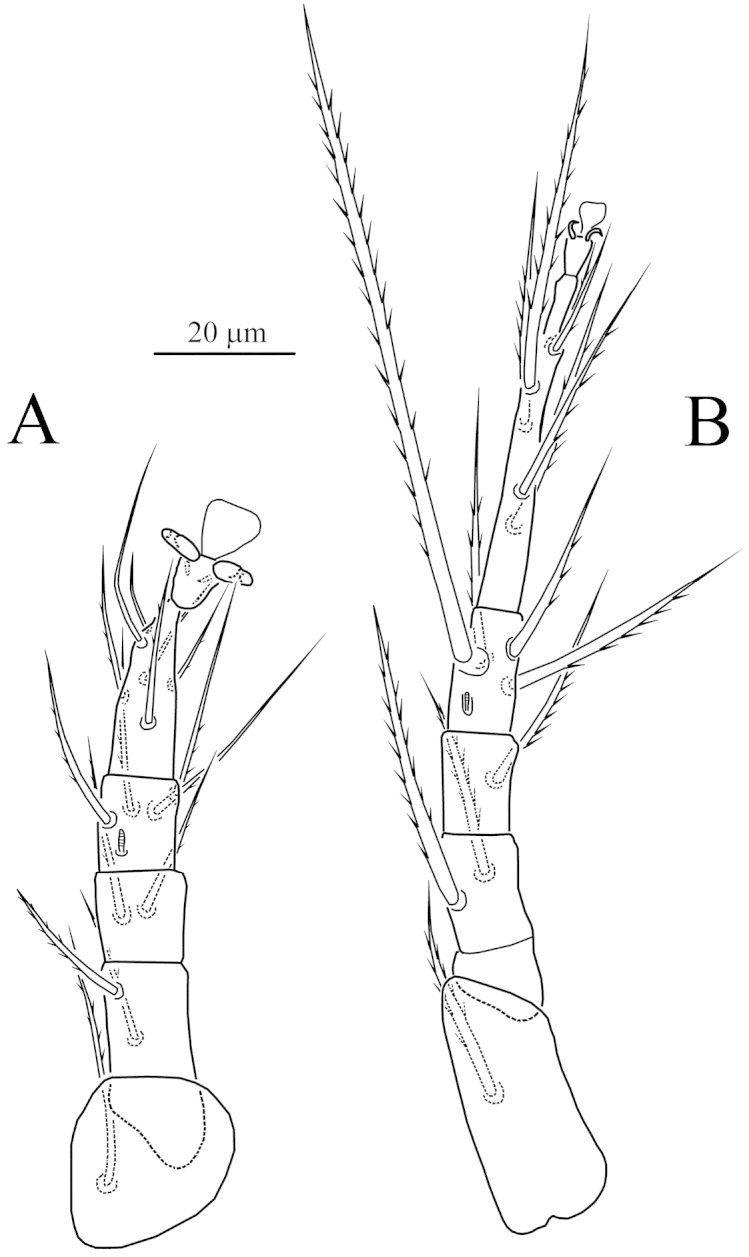
*Caesarodispus samsinaki* (Mahunka, 1967), comb. n., female: **A** leg III **B** leg IV.

Legs (Figs 6–7). Leg I (Fig. 6A) distinctly shorter and thinner than leg II. Setal formula: 1–3–4–16(3). Tibiotarsus not thickened, cylindrical. Length of solenidia *ω*_1_ 11 > *φ*_1_ 5 < *φ*_2_ 10; *ω*_1_ and *φ*_2_ finger-shaped, *φ*_1_ clavate, solenidion *ω*_2_ absent. Seta *k* very long, slightly longer than combined genu and tibiotarsus I. Leg II (Fig. 6B). Setal formula: 1–2–3–4(1)–6(1). Tarsus with sickle-like, padded claws and large empodium. Solenidion *ω* 8, finger-shaped, solenidion *φ* 4 weakly clavate. Setae *v*” of femur II absent. Leg III (Fig. 7A). Setal formula: 1–2–2–4(1)–6. Claws of same shape as on tarsus II. Solenidion *φ* 4 weakly clavate. Seta *dFe* blunt-ended. Leg IV (Fig. 7B). Setal formula: 1–2–1–4(1)–6. Tarsus long and thin, pretarsus short, with two small simple claws and small empodium. Solenidion *φ* 4, weakly clavate. Seta *dFe* distinctly blunt-ended.

**Male** and **larva** unknown.

##### Material examined.

One female, **Russia:** Tyumen Province, vicinity of Tyumen, 57°04'03"N, 65°04'12"E, on ants *Formica rufa* L., 17 August 2014, coll. A.A. Khaustov.

##### Distribution and hosts.

This species was originally described from the Czech Republic (Mahunka 1967) where it was collected from ants *Formica rufa*. It was also recorded from Ukraine and Belarus (Sevastianov 1978). This is a new record for the fauna of Russia.

##### Remarks.

Mahunka (1967) described this species in the genus *Pygmephorus* Kramer (Pygmephoridae). Later on, he replaced it to the genus *Brennandania* Sasa, 1961 (Microdispidae) (Mahunka 1972). Sevastianov (1978) placed this species in the genus *Petalomium* Cross, 1965 (Neopygmephoridae).

According to key to families of the superfamily Pygmephoroidea provided by Khaustov and Ermilov (2011) this species undoubtedly belongs to the family Microdispidae by having three setae on femur I, unmodified seta *dFeI* and presence of only one pair of setae on prodorsum. I placed this species in the genus *Caesarodispus* based on the following combination of characters: 1) tibiotarsus I without claw, 2) leg I distinctly shorter and thinner than leg II, 3) seta *s* of tibiotarsus I present, 4) pharyngeal pumps 1 and 3 vestigial, pharyngeal pump 2 large, distinctly transversely striated, 5) posterior margin of posterior sternal fig entire. All of these characters well agree with diagnosis of the genus *Caesarodispus*.

Unlike other known species of the genus *Caesarodispus*, *Caesarodispus samsinaki* has only two setae on femur II (*v*” absent) and only three solenidia on tibiotarsus I (*ω*_2_ absent). However reduction of leg chaetotaxy was also recorded in C. *klepzigi* Khaustov and Moser, 2008, which has only two setae on femur I, while other *Caesarodispus* species have three setae.

The original description of *Caesarodispus samsinaki* of Mahunka (1967) is incomplete, thus I made a redescription of this species. The present redescription of *Caesarodispus samsinaki* is based on material from Western Siberia. The specimens from Western Siberia are well agreed with original description of *Caesarodispus samsinaki*, especially having lanceolate dorsal idiosomal setae and unique reticulated hysterosomal tergites, and undoubtedly conspecific with it.

#### Caesarodispus minutus

Taxon classificationAnimaliaProstigmataMicrodispidae

(Sevastianov, 1981)

Microdispus minutus Sevastianov, 1981, p. 28, fig. 5.
Caesarodispus minutus : Khaustov 2008: 390.

##### Material examined.

Eleven females, Russia: Tyumen Province, Tyumen region, vicinity of settlement Narimanovo, 57°21'56"N, 65°08'21"E, in the nest of ants *Lasius flavus* (Fabricius), 30 July 2014, coll. V.A. Stolbov.

This species was described from Ukraine from ants *Tetramorium caespitum* L. (Sevastianov 1981). Khaustov (2009) redescribed it based on type material. It was also recorded from Iran from *Temnothorax* sp. (Loghmani et al 2014). This is a new record for the fauna of Russia.

#### Key to world species of the genus *Caesarodispus* (females)

**Table:** 

1	Hysterosomal tergites not reticulated, femur II with three setae	**2**
–	Hysterosomal tergites distinctly reticulated, femur II with two setae (*v*” absent)	***Caesarodispus samsinaki* (Mahunka, 1967), comb. n.** Czech Republic, Ukraine, Belarus, Russia. On *Formica rufa*.
2	Seta *v*’ of genu I not thickened, pointed	**3**
–	Seta *v*’ of genu I distinctly thickened, widened distally, strongly barbed	***Caesarodispus pusillus* Khaustov, 2009.** Crimea. In the nest of *Crematogaster schmidti*
3	Seta *d* no more than 1.5 times longer than *f*	**4**
–	Seta *d* about 4 times longer than *f*	***Caesarodispus klepzigi* Khaustov & Moser, 2008.** U.S.A. On *Solenopsis invicta*
4	Seta *d* of tibia IV heavily barbed, reaching beyond tip of pretarsus IV	**5**
–	Seta *d* of tibia IV smooth or weakly barbed, not reaching beyond tip of pretarsus IV	**7**
5	Seta *d* of femur IV subequal to or longer than *tc*” of tarsus IV	**6**
–	Seta *d* of femur IV more than 2 times shorter than *tc*” of tarsus IV	***Caesarodispus gaius* Mahunka, 1977.** France. On *Myrmica sabuleti*
6	Setae *d* and *f* lanceolate and strongly barbed	***Caesarodispus brevipes* Mahunka, 1986.** Hungary. In ant nest
–	Setae *d* and *f* not lanceolate, weakly barbed	***Caesarodispus modestus* (Berlese, 1903).** Italy, Russia (Crimea). On *Messor* spp.
7	Posterior part of aggenital fig smooth	**8**
–	Posterior part of aggenital fig distinctly reticulated	***Caesarodispus shandizensis* Loghmani & Hajiqanbar, 2014.** Iran. On *Temnothorax* sp.
8	Setae *f* distinctly longer than distance *f*–*f*	***Caesarodispus minutus* (Sevastianov, 1981).** Ukraine, Iran, Russia. On *Tetramorium caespitum*, *Lasius flavus*, *Temnothorax* sp.
–	Setae *f* shorter than distance *f*–*f*	***Caesarodispus acuminatus* (Sevastianov, 1981).** Ukraine. On *Tetramorium caespitum*

## Supplementary Material

XML Treatment for Unguidispus

XML Treatment for Unguidispus polyctenus

XML Treatment for Unguidispus lasii

XML Treatment for Unguidispus japonicus

XML Treatment for Caesarodispus

XML Treatment for Caesarodispus samsinaki

XML Treatment for Caesarodispus minutus

## References

[B1] Berlese A (1903) Diagnosi di alcune nuove specie di Acari italiani, mirmecofili e liberi. Zoologischer Anzeiger 27: 12–28.

[B2] Cross EA (1965) The generic relationships of the family Pyemotidae (Acarina: Trombidiformes). The University of Kansas science bulletin 45: 29–275.

[B3] Grandjean F (1944) Observations sur les Acariens de la famille des Stigmaeidae. Archives des Sciences Physiques et Naturelles 5, 26: 103–131.

[B4] Grandjean F (1947) L’origine pileuse des mors et la chaetotaxie de la mandibule chez les Acariens actinochitineux. Comptes rendus des séances de l'Academie des Sciences 224: 1251–1254.

[B5] Hajiqanbar H Hosseininaveh F (2014) A new genus and species of the family Microdispidae (Acari: Prostigmata) associated with *Oryctes nasicornis* (Coleoptera: Scarabaeidae) and redescription of the monotypic genus *Vietodispus* Mahunka, 1975. Zoological Studies 53: 58–70. doi: 10.1186/s40555-014-0058-7

[B6] Kaliszewski M Athias-Binche F Lindquist EE (1995) Parasitism and parasitoidism in Tarsonemina (Acari: Heterostigmata) and evolutionary considerations. Advances in Parasitology 35: 335–367. doi: 10.1016/S0065-308X(08)60074-3 7709855 10.1016/s0065-308x(08)60074-3

[B7] Khaustov AA (2004) Mites of the family Neopygmephoridae Cross, 1965 stat. n. and their position in Heterostigmata. In: Balashov YS (Ed.) VIII Russian Acarological Conference, St.-Petersburg Zoological Institute of RAS, St.-Petersburg, 137 [in Russian]

[B8] Khaustov AA (2008) Mites of the family Scutacaridae of Eastern Palaearctic. Akademperiodyka, Kiev, 291 pp.

[B9] Khaustov AA (2009) New and little known species of mites of the genus *Caesarodispus* (Acari, Heterostigmata, Microdispidae) associated with ants (Hymenoptera, Formicidae) from Ukraine. Vestnik Zoologii 43: 387–393 http://www.degruyter.com/view/j/vzoo.2009.43.issue-5/v10058-009-0017-7/v10058-009-0017-7.xml

[B10] Khaustov AA Ermilov SG (2011) A new species of the genus *Siteroptes* (Acari: Heterostigmata: Pygmephoridae) from European Russia. Entomological Review 91(4): 528–532. doi: 10.1134/S0013873811040178

[B11] Khaustov AA Moser JC (2008) Two new species of mites of the genera *Petalomium* Cross and *Caesarodispus* Mahunka (Acari: Heterostigmata: Neopygmephoridae, Microdispidae) associated with *Solenopsis invicta* Buren (Hymenoptera: Formicidae) from the U.S.A. International Journal of Acarology 34: 115–121. doi: 10.1080/01647950808683714

[B12] Kurosa K (1979) Three new species of *Unguidispus* (Acari, Heterostigmata,Microdispidae) from Japan. Annotationes Zoologicae Japonenses 521: 63–71.

[B13] Lindquist EE (1986) The world genera of Tarsonemidae (Acari: Heterostigmata): a morphological, phylogenetic, and systematic revision, with a reclassification of family-group taxa in Heterostigmata. Memoirs of the Entomol. Society of Canada 136: 1–517. doi: 10.4039/entm118136fv

[B14] Loghmani A Hajiqanbar H Talebi AA (2014) New species and new record of the genus *Caesarodispus* (Acari: Heterostigmatina: Microdispidae) phoretic on *Temnothorax* sp. (Hymenoptera: Formicidae). Annales Zoologici 64(2): 273–278. doi: 10.3161/000345414X680627

[B15] Mahunka S (1967) Beiträge zur Kenntnis der Tschechoslowakischen Tarsonemini-Fauna. Věstník Československé společnosti zoologické 31: 240–244.

[B16] Mahunka S (1968) *Xystrorostrum* gen. n. und eine neue *Siteroptes*-art aus Ungarn (Acari). Reichenbachia 10: 127–131.

[B17] Mahunka S (1970a) Considerations on the systematic of the Tarsonemina and the description of new European taxa (Acari: Trombidiformes). Acta Zoologica Hungarica 16: 137–174.

[B18] Mahunka S (1970b) Beiträge zur Kenntnis der Milbenfauna der Ötztaler Alpen. 1. Tarsoneminen-Arten aus der Umgebung von Obergurgl. Opuscula Zoologica (Budapest) 10: 271–289.

[B19] Mahunka S (1972) Tetűatkák - Tarsonemina (Magyarország állatvilága – Fauna Hungariae 110.) – XVIII. kötet, 16. füzet (Arachnoidea). Akademiai Kiado, Budapest, 215 pp.

[B20] Mahunka S (1977a) Neue und interessante Milben aus dem Genfer Museum XiX. Einige Angaben zur Kenntnis der Milbenfauna der Ameisen-Nester (Acari: Acarida, Tarsonemida). Archives des Sciences Geneve 30: 91–106.

[B21] Mahunka S (1977b) The examination of myrmecophilous tarsonemid mites based on the investigations of Dr. c. w. rettenmeyer (Acari). Acta Zoologica Academiae Scientiarum Hungaricae 23: 99–132.

[B22] Mahunka S (1987) Tarsonemids of the Kiskunság National park (Acari). In: Mahunka S (Ed.) The fauna of the Kiskunság National park, 1 Akademiai Kiado, Budapest, 435–455.

[B23] Sasa M (1961) New mites of the genus *Pygmephorus* from small mammals in Japan (Acarina: Pyemotidae). Japanese Journal of Experimental Medicine 31: 191–208. 14497311

[B24] Sevastianov VD (1969) New genus and species of mites of the Pyemotidae (Trombidiformes) family and their position in the family. Vestnik Zoologii 3: 66–71. [in Russian]

[B25] Sevastianov VD (1978) Tarsonemina. In: Ghilarov MS (Ed.) Opredelitel pochvoobitayushchikh kleshchey. Trombidiformes, Nauka, Moscow, 14–90 [in Russian]

[B26] Sevastianov VD (1981) New species of mites of the family Pygmephoridae (Tarsonemina, Trombidiformes). Vestnik Zoologii 6: 25–29 [in Russian]

